# Impact of Cryopreserved Placental Allografts on Biochemical Recurrence in Prostate Cancer

**DOI:** 10.3390/cancers16172973

**Published:** 2024-08-26

**Authors:** Josh Gottlieb, Douglas A. Hanes, Matias A. Bustos, Jane Choe, Albert Luu, Daniel Seizer, Dave S. B. Hoon, Timothy G. Wilson

**Affiliations:** 1 Department of Urologic Oncology, Providence Saint John’s Cancer Institute, Santa Monica, CA 90404, USA; jachoe@bu.edu (J.C.); albert.luu@md.cusm.edu (A.L.); daniel.seizer@providence.org (D.S.); timothy.wilson@providence.org (T.G.W.); 2 Department of Biostatistics, Providence Saint Joseph Health Center, Portland, OR 97213, USA; douglas.hanes@providence.org; 3 Department of Translational Molecular Medicine, Providence Saint John’s Cancer Institute, Santa Monica, CA 90404, USA; matias.bustos@providence.org (M.A.B.); dave.hoon@providence.org (D.S.B.H.)

**Keywords:** prostate cancer, prostatectomy, biochemical recurrence-free survival, BCR, placental allograft

## Abstract

**Simple Summary:**

Placental allograft tissue has been extensively used in wound healing. It can also be placed along the neurovascular bundles during radical prostatectomy to improve post-operative continence and erectile function recovery. The existing literature shows how placental allografts lead to improved functional recovery after radical prostatectomy. It is unknown whether these allografts have an impact on biochemical recurrence (BCR). Our study was a single surgeon retrospective review of 362 radical prostatectomy cases. The subgroups were the negative control group and two brands of cryopreserved amniotic membrane (CAM). Our data show improved continence recovery using CAM allografts. At a median follow-up of 41 months, there was no observed impact of BCR. Interestingly, in vitro analysis also revealed decreased cell viability of prostate cancer cell lines when incubated with the CAM allograft. Although further investigation is required, our data do not support a need for oncologic concern while using CAM allografts during radical prostatectomy.

**Abstract:**

Background: Human placental allografts are widely used to promote wound healing. Placental (or amniotic membrane/umbilical cord) allografts are placed along the neurovascular bundles during radical prostatectomy to improve continence and erectile function recovery. It is unknown whether placental allografts impact biochemical recurrence (BCR). Methods: This was a single-surgeon retrospective study of 566 robotic radical prostatectomies performed from April 2015 to March 2021. The patients were divided into three groups: the negative control, Brand A, and Brand B. Brand A and Brand B were both cryopreserved amniotic membrane (CAM) allografts. A total of 324 cases were included for BCR Kaplan–Meier and risk-adjusted multivariate analyses (362 for continence analysis). In vitro analyses were performed to determine the effect of CAM allografts on prostate cancer (PCa) cell line growth. Results: For propensity score-matched analysis (adjusting for pre-operative PSA, tumor stage, Gleason Grade, and margin status), (1) the allograft groups did not show differences in time to BCR vs. the negative control group (*p* = 0.7), and (2) combined allograft treatment groups showed better continence recovery vs. the negative controls (*p* = 0.01). In vitro, placental allografts reduced PCa cell line growth in co-culture assays. Conclusions: cryopreserved AM allografts (combined or individual brands) did not show a significant effect on BCR but improved continence recovery for PCa patients.

## 1. Introduction

Prostate cancer (PCa) is the second most common cancer in men worldwide, with over 1.4 million new cases reported in 2020 [1]. Standard treatments for localized PCa have historically involved radiation therapy (RT) with or without androgen deprivation therapy (ADT) or radical prostatectomy with or without pelvic lymph node dissection [2]. Over the last few decades, radical prostatectomy techniques have quickly evolved in the hopes of improving patient outcomes. The transition from open surgery to robotic-assisted radical prostatectomy (RARP) improved clinical parameters such as intraoperative blood loss, intraoperative transfusion rates, and length of hospital admissions [3]. However, two common side effects after any prostatectomy approach are erectile dysfunction and urinary incontinence. As such, many surgical techniques have been developed over the years to hasten the recovery of continence and erectile function after RARP. For example, recent innovative techniques like retzius-sparing and extraperitoneal, transperineal, and transvesical approaches were developed utilizing both multi- and single-port robotic platforms [4]. Another technique aimed at improving continence and erectile function involves the use of human placenta-derived allografts, which can involve amniotic membranes (AM), chorionic membranes (CM), and the umbilical cord (UC) [5].

For several decades, human placenta-derived allografts have been utilized for their therapeutic benefit by reducing inflammation and promoting regenerative healing [6]. These allografts have been used in ocular surgery [7,8], tendon repair [9], wound healing [10,11], and burns [12,13]. AM has also been shown to support nerve regeneration. Studies have demonstrated more axons with myelin sheaths of normal thickness and less inter-axonal fibrosis when treated with AM [14,15].

The concept of utilizing placenta-derived allografts in prostatectomy patients is to improve the time to continence and sexual potency by placing them along the neurovascular bundle (NVB) intraoperatively. Evidence supports that the allografts regulate inflammation, improve healing, and recruit local stem cells, which collectively allow for improved continence and potency outcomes [16,17,18,19,20].

Despite the evidence supporting improved continence and erectile function recovery after prostatectomy, there is still uncertainty about whether placental allografts have an impact on biochemical recurrence-free survival (BRFS). Some hypothesize that recruiting local mesenchymal stem cells into a cancer field is a risk factor for cancer recurrence, which is supported by a study conducted by Alvim et al. at Memorial Sloan Kettering Cancer Center (MSKCC). The authors claimed that there were faster tumor relapse and growth in the allograft groups compared to control groups in in vitro and in vivo models [21]. However, there are also data claiming that placental stem cells may have a protective effect on cancer control [22]. In addition, another review states that placental allografts improve functional recovery without impacting oncological control [17].

With the lack of conclusive evidence regarding the impact of placental allografts on BRFS, we sought to analyze our extensive institutional experience using cryopreserved AM allografts during RARP. We hypothesized that cryopreserved AM allografts improve continence recovery and have no impact on BRFS. We did not seek to analyze erectile function outcomes due to inconsistencies in erectile function documentation before and after RARP.

## 2. Materials and Methods

### 2.1. Study Population

This study was performed in accordance with the institutional review board protocol of Providence St. John’s Health Center (PSJHC). Clinical and pathological information was retrospectively collected via electronic medical record (EMR) from 566 patients after bilateral nerve-sparing RARP with or without extended pelvic lymph node dissection (ePLND) performed by a single surgeon from April 2015 to March 2021. The negative control group included the cases without any allograft placed, which occurred from April 2015 to June 2017. Starting in June 2017, cryopreserved AM allografts were utilized in every case as standard practice for the surgeon. The two allograft brands, Brand A and Brand B, were randomly alternated from June 2017 to December 2019. Both allograft brands were cryopreserved/processed similarly and did not contain any living stem cells. Starting in December 2019, the surgeon exclusively used Brand A. Aside from the allograft used, the surgical technique remained unchanged throughout the study timeline.

Outcome data were obtained from the PSJHC EMR, which included available records from outside institutions. Patient demographics, pathological data, and clinical data (BCR, continence, potency, disease/survival status, need for adjuvant/salvage therapy, etc.) were recorded. Post-operative PSA values used for this study were the following: first post-operative PSA (approximately 6 weeks after surgery), PSA at time of BCR, and PSA at most recent follow-up.

### 2.2. Definitions

Disease status at most recent follow-up was determined by most recent PSA (PSA < 0.2 ng/mL = patient without disease; PSA ≥ 0.2 ng/mL = disease present). Continence was defined as when a patient used one safety pad per day or no pads per day.

### 2.3. Inclusion/Exclusion Criteria

Patients were excluded from this study for the following reasons: (1) persistent post-operative PSA (defined by detectable PSA > 0.1), (2) neoadjuvant hormonal therapy, (3) cytoreductive or salvage RARP, (4) less than 1-year follow-up data, and (5) lymph node metastasis (only excluded for BRFS analysis).

After reviewing the initial 566 patients, 324 patients met inclusion criteria for BRFS analysis, and 362 patients met inclusion criteria for continence analysis.

### 2.4. Study End Points:

The primary outcome measure was BRFS, where BCR was defined by a post-operative PSA of ≥0.2 ng/mL after an initially undetectable post-operative PSA. Secondary outcome was urinary continence recovery. Due to a lack of consistent perioperative documentation and various types of erectile function surveys utilized, potency recovery was not included in the analysis to avoid misinterpretation of statistical results.

### 2.5. Prostate Cancer Cell Lines

Established prostate cancer cell lines (LNCap and DU145) were obtained from the American Type Culture Collection (ATCC). DU145 cell line was cultured in DMEM medium supplemented with 10 mM HEPES, 10% heat-inactivated fetal bovine serum (FBS), and 1% penicillin-streptomycin. LNCap cell line was cultured in RPMI-1640 medium supplemented with 10 mM HEPES, 10% FBS, and 1% penicillin-streptomycin. All the above cell lines were authenticated using short tandem repeat (STR) profiling and were mycoplasma-free.

### 2.6. Cell Proliferation Assay

LNCap and DU145 cell lines (2 × 10^4^ cells/well) were seeded in 24-well plates (Thermo Fisher Scientific, Waltham, MA, USA) and incubated for 24 h in the respective culture medium. Then, LNCap and DU145 cell lines were co-incubated with a section of 25 mm^2^ placental allograft membrane for 0, 24, and 48 h. Also, DU145 cell lines were co-incubated with different sizes of placental allograft membranes (25 mm^2^, 12.5 mm^2^, and 6.25 mm^2^) and incubated for 24 h. Additionally, different sizes of placental allograft membrane (25 mm^2^, 12.5 mm^2^, and 6.25 mm^2^) were placed in a Boyden chamber, which was then placed inside the 24-well plate and incubated for 24 h. After the indicated times, the placental allograft membrane or Boyden chambers containing the placental allograft membrane were removed and cell viability was measured using a Cell Titer-Glo Luminescent Cell Viability assay with a GloMax-MultiDetection System (Promega, Madison, WI, USA) according to the manufacturer’s instructions and as previously described [23,24]. The relative cell proliferation was calculated by measuring the number of viable cells at the indicated time points. The values obtained at the designated times were normalized to control conditions.

### 2.7. Statistical Analysis—Clinical Data

Differences in time to BCR and time to recovery of continence were compared between treatment groups using Kaplan–Meier analyses of both complete available cohorts and of groups matched 1:1 on covariates, including pre-surgery PSA (log-transformed), pathological stage (pT1/pT2 vs. pT3), Gleason Grade Group (1–5), surgical margins (R0 vs. R1), and nodal involvement (N0 vs. N1 vs. NX). Due to potential confounding between treatment type and prognostic factors, we considered the results of matched analyses to be primary. Primary comparisons are between allograft treatment (with either product) and the control; however, we also conducted sensitivity analyses to assess for variation in results dependent upon the specific product used.

Matching 1:1 was applied separately for each pair of treatment groups to be compared; however, analyses of BCR and recovery of continence use the same matched groups, with limited reduction in sample size due to missing data for recovery of continence. We obtained satisfactory matching for the main comparison of allograft treatment to control, with all 143 control patients matched to 143/219 allograft patients and absolute values of standardized mean differences less than 0.07 for all component matching factors.

Matching was carried out using the R package MatchIt, with the “optimal” method. All analyses were completed using R v.4.2.2. Statistical significance was defined as *p* < 0.05.

### 2.8. Statistical Analysis—Cell Culture Data

All the statistical analyses were performed using GraphPad Prism 8 software (GraphPad Software Inc., La Jolla, CA, USA). Statistical significance was calculated by using T-test or One-Way ANOVA test and post hoc test. A two-sided *p* < 0.05 was considered statistically significant: *p* < 0.05, ** *p* < 0.01, *** *p* < 0.001, *** *p* < 0.0001, and ns = not significant. All figures were unified using Adobe Illustrator CC (Adobe, San Jose, CA, USA) or CorelDraw graphics suite 8X (Corel, Ottawa, Canada).

## 3. Results

A total of 362 patients who underwent bilateral nerve-sparing RARP with or without cryopreserved AM allograft met inclusion criteria for retrospective analysis. The three subgroups consisted of the negative control group (n = 143), Brand A (n = 142), and Brand B (n = 77). Patient clinical and pathological data from the overall cohort and subgroups are summarized in Table 1. The mean age at the time of surgery was 64.9 years (standard deviation (SD) ± 7.4 years) with a median follow-up time of 41 months (interquartile range (IQR) 27–56 months). The mean preoperative PSA was 8.2 ± 6.9 ng/mL and the median post-operative Gleason Grade was 2 (IQR 2–3). A total of 62% of patients underwent ePLND, of which 83% were pN0. Positive surgical margins were seen in 37% of patients.

The postoperative clinical outcomes of the overall cohort and subgroups are summarized in Table 1. At a median follow-up time of 41 months, 358 patients (99%) in the overall cohort were alive at the most recent follow-up (90% alive without disease, 9% alive with disease). Only four patients expired (three without disease present, one with disease present). BCR was observed in 72 patients (20%). Continence was achieved in 329 patients (93%) in the overall cohort, 125 (91%) in the control group, 133 (96%) in the Brand A group, and 71 (93%) in the Brand B group. The median pads per day at the last follow-up were 0 (IQR 0) across the overall cohort and subgroups.

**Figure 1:**
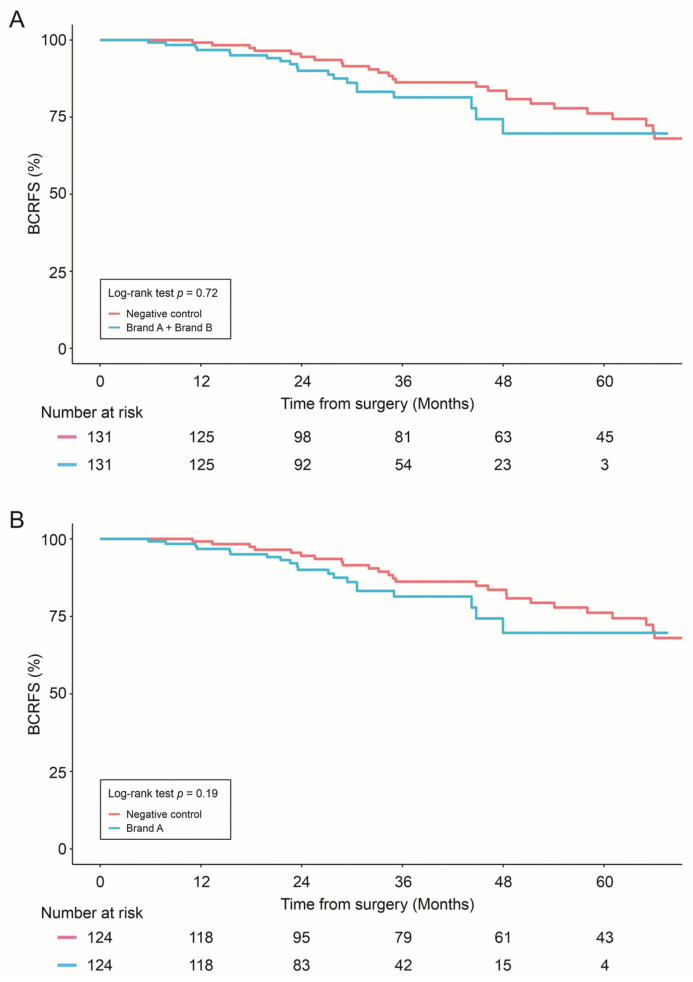

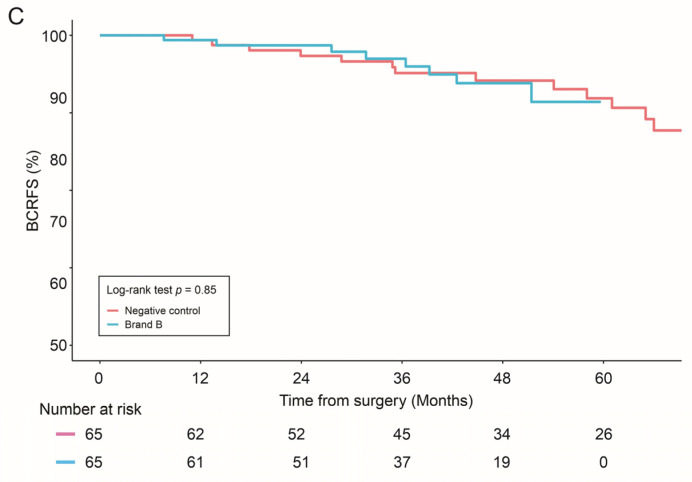
Biochemical Recurrence-free survival (BCRFS) was compared in: Negative control vs. combined allograft groups (Brand A + Brand B) (**A**); Negative control vs. Brand A allograft group (**B**); and Negative control vs. Brand B allograft group (**C**). Statistical differences were analyzed by Log-rank test.

For propensity score-matched analyses, no statistically significant difference in BRFS was observed between combined allograft groups (Brand A + Brand B) vs. negative control (*p* = 0.72), individual allografts (Brand A or Brand B) vs. negative control (*p* = 0.19 and *p* = 0.85, respectively), or between the allografts themselves (Brand A vs. Brand B, *p* = 0.38) (Figure 1).

**Figure 2:**
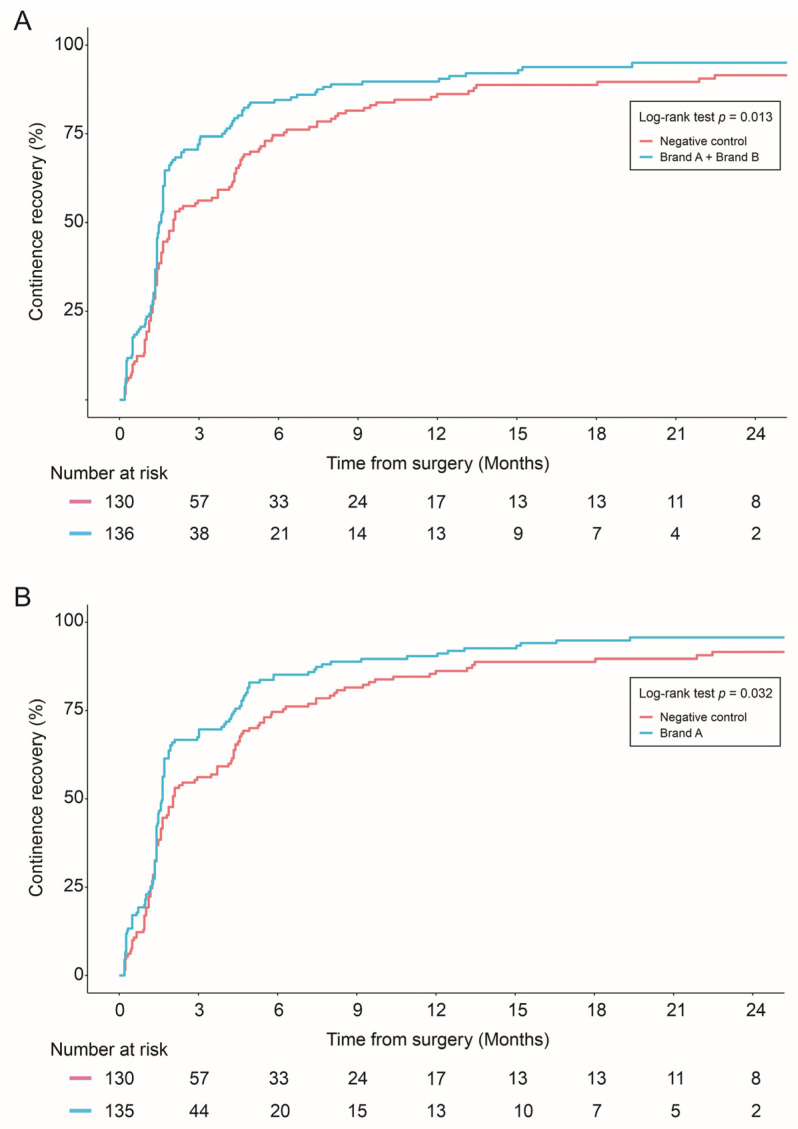

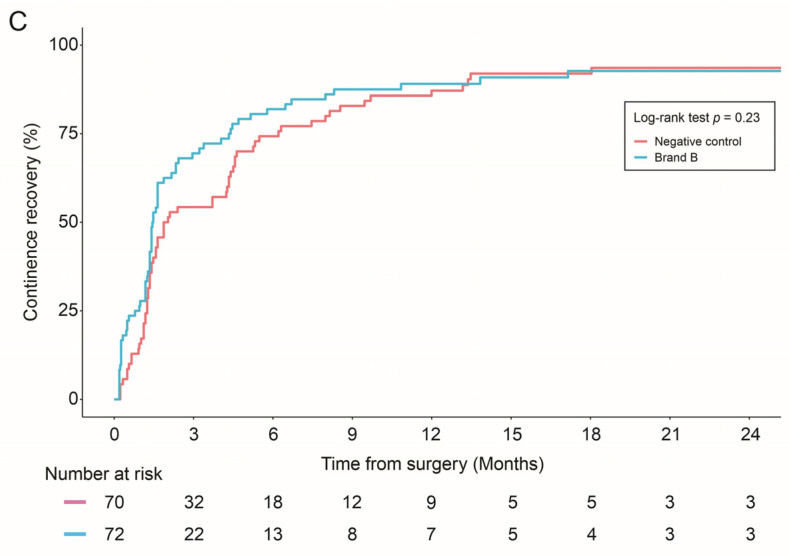
Continence recovery outcomes were compared in: Negative control vs. combined allograft groups (Brand A + Brand B) (**A**); Negative control vs. Brand A allograft group (**B**); and Negative control vs. Brand B allograft group (**C**). Statistical differences were analyzed by Log-rank test.

The combined allograft group and Brand A alone showed improved continence recovery compared to the control group: (1) Brand A + Brand B vs. negative control (*p* = 0.01), (2) Brand A vs. control (*p* = 0.03) (Figure 2).

### In Vitro Prostate Cancer Cell Growth Assay

**Figure 3:**
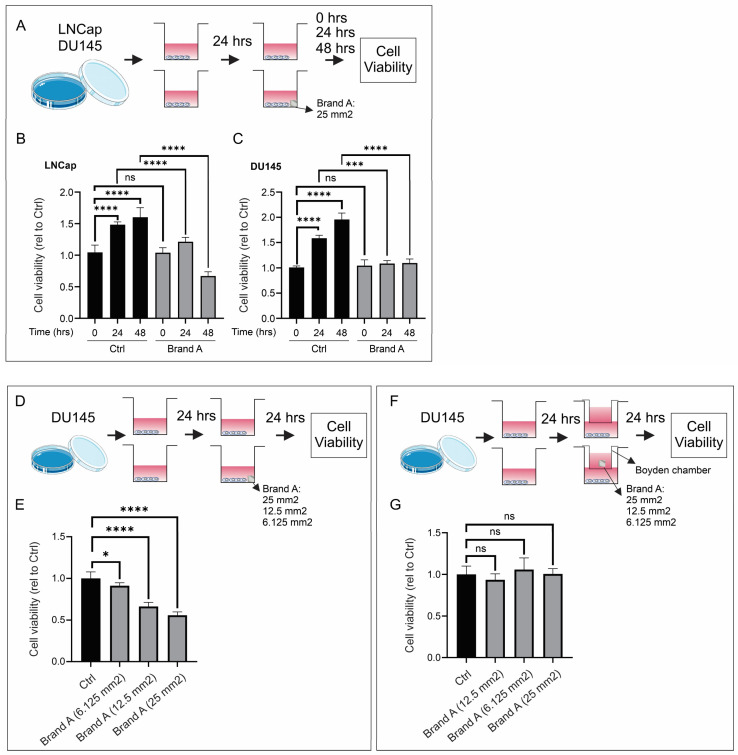
In vitro prostate cancer cell growth assay. (**A**–**C**) Addition of placental allograft membrane significantly decreased cell viability after 24 and 48 h of incubation. (**D**,**E**) Inhibition of cell viability was dependent on size of placental allograft membrane. (**F**,**G**) Varying size of placental allograft membranes did not inhibit cell viability when assessing if secreted molecules were responsible for cell growth inhibition. ns: non-significant, * *p* < 0.05, *** *p* < 0.001,**** *p* < 0.0001.

Prostate cancer cell lines (LNCap and DU145) were incubated with a section of Brand A placental allograft membrane (25 mm^2^) to evaluate the influence on cell viability at different time points (0, 24, and 48 h). While both PCa cell lines grew under control conditions, the addition of the placental allograft membrane significantly decreased cell viability after 24 and 48 h of incubation (Figure 3A–C). Of note, the inhibition of cell viability depended on the size of the placental allograft membrane (Figure 3D,E). Finally, placental allograft membranes were placed in Boyden chambers and then incubated with PCa cell lines to determine if the inhibition of cell growth was mediated by secreted molecules. Under these conditions, placental allograft membranes did not inhibit cell viability (Figure 3F,G). To summarize, in vitro placental allograft membranes decreased the growth of PCa cell lines.

## 4. Discussion

Despite the advances in operative technique and technology seen over the last decade, surgeons still seek to improve clinical outcomes following RARP. The use of human placenta-derived allografts is another attempt at improving continence and potency recovery. Their use in prostatectomy patients is due to the existing research showing that these allografts regulate inflammation, improve healing, and may also improve nerve regeneration [10,12,14,15]. This effect is thought to be from the ability of the allograft to induce apoptosis of neutrophils, monocytes, and macrophages while also reducing the infiltration of neutrophils, macrophages, and lymphocytes [25,26,27]. Placental allograft tissue also has extensive amounts of cytokines and neurotrophic factors, like nerve growth factors, that are involved in nerve regeneration and epithelial healing [19,28,29,30]. In addition, a matrix compound that is reportedly found in Brand A called heavy chain-hyaluronic acid/pentraxin 3 (HC-HA/PTX3) has also been shown to exert anti-inflammatory effects [26].

Data already exist showing improved continence and erectile function recovery following RARP [16,17,18,19,20]. In a retrospective analysis by Dr. Ahmed et. al, umbilical cord allografts were found to have a significant favorable effect on continence at 1 month, 3 months, and 12 months after RARP compared to the negative controls [19].

However, despite the functional benefits of using placental allografts, there is not a clear understanding as to whether this may clinically impact oncological control. The study conducted at MSKCC assessed the impact of dehydrated human amnion/chorion membrane (dHACM) allografts on prostate and bladder cancer growth in the setting of residual disease and positive surgical margins. Using in vitro cancer cell lines and in vivo mouse models (where tumors were partially resected to represent positive surgical margins), they concluded that there was faster tumor relapse and growth in the allograft groups [21]. Contrary to this claim, a study conducted by Peak et al. in 2018 reported that exosomes secreted by placental stem cells selectively inhibit the growth of aggressive prostate cancer cells [22]. In addition, a recent literature review claimed that placental allografts improved functional outcomes after RARP without impacting oncological control [17].

Our data support the claim that there is no increased risk for BCR when using placental allografts, while still showing the benefit of earlier continence recovery. Improved continence recovery was observed in the combined allograft treatment group and Brand A group alone when compared to negative controls. We believe the reason why Brand B alone did not show improved continence recovery may be due to a smaller sample size (n = 77) compared to the Brand A group (n = 142).

Furthermore, our in vitro cell viability assay suggests a possible inhibitory effect on the growth of PCa cell lines. However, our study only investigated cell viability up to 48 h. The observed decrease in PCa cell viability with allograft membranes would have to be further investigated at longer time points. A possible explanation for the different results obtained in our study compared to the MSKCC study may be due to the processing method of different allografts. MSKCC used dehydrated allografts (dHACM), whereas our study used devitalized cryopreserved allografts. As a result, the extracellular matrix and its contents may vary due to the allograft processing methods.

One limitation of our study is the retrospective nature and the lack of randomization between allograft brands and negative controls. There was also a noticeable sample size discrepancy between the two allograft groups (Brand A, n = 142 and Brand B, n = 77). As mentioned, this may explain why Brand B did not show improved continence recovery like Brand A and the combined group. However, regarding oncological control, we do not believe the sample size discrepancy was as relevant since all study groups (combined group, Brand A, and Brand B) did not show increased risk for BCR. Although propensity score matching was performed, we still acknowledge that the different sample sizes between treatment groups could affect our study conclusions. Another limitation of our study is the increased surgeon experience over time, which could potentially impact clinical outcomes despite the unchanged surgical technique. Like many studies, there is also a lack of long-term follow-up for both our clinical and in vitro analyses. We felt that the median follow-up time of 41 months was acceptable because the majority of BCRs occur within 3 years after prostatectomy and >95% of patients achieve continence within 12 months after prostatectomy.

## 5. Conclusions

In this single-surgeon retrospective analysis, cryopreserved AM allografts did not appear to have an impact on BRFS at a median follow-up time of 41 months. The combined allograft treatment groups and Brand A group alone both showed improved continence recovery compared to the control group. In vitro analysis revealed decreased cell viability of PCa cell lines at 24 and 48 h when incubated with cryopreserved AM allografts. Further research is needed to assess if human placenta-derived allografts have any impact on BRFS and oncological outcomes.

## Figures and Tables

**Table 1:** Clinical outcomes/features comparing negative controls and cryopreserved amniotic membrane allograft groups (Brand A, Brand B).

	Overall Cohort	Negative Control	Brand A	Brand B
Number of Patients in Each Group	362	143	142	77
Median (IQR) Follow-up Time	41 months (27–56 months)			
Survival Status at Most Recent Follow-up				
Alive without disease	325 (90%)	126 (88%)	133 (94%;)	66 (85%)
Alive with disease	33 (9%)	16 (11%)	7 (5%)	10 (13%)
Deceased without disease	3 (1%)	1 (1%)	2 (1%)	0
Deceased with disease	1 (0.3%)	0	0	1 (1%)
BCR				
Yes	72 (20%)	32 (22%)	27 (19%)	13 (17%)
No	290 (80%)	111 (78%)	115 (81%)	64 (83%)
Any Salvage Therapy Given (ADT, RT, and/or chemo)	70 (20%; N = 358)	35 (25%; N = 141)	27 (19%; N = 142)	8 (12%; N = 75)
Salvage ADT	46 (66%; N = 70)	21 (60%; N = 35)	18 (67%; N = 27)	7 (88%; N = 8)
Salvage RT	62 (89%; N = 70)	33 (94%; N = 35)	22 (82%; N = 27)	7 (88%; N = 8)
Salvage Chemotherapy	9 (3%; N = 70)	9 (26%; N = 35)	0	0
Post-operative Continence Achieved (defined as ≤1 security pad per day)				
Yes	329 (93%; N = 352)	125 (91%; N = 137)	133 (96%; N = 139)	71 (93%; N = 76)
No	23 (7%; N = 352)	12 (9%; N = 137)	6 (4%; N = 139)	5 (7%; N = 76)
N/A	10 (3%; N = 362)	6 (4%; N = 143)	3 (2%; N = 142)	1 (1%; N = 77)
Median (IQR) post-operative Gleason Grade	2 (2–3)	2 (2–3)	3 (2–3)	2 (2–3)
Pathological T Stage				
pT1	3 (1%)	3 (2%)	0	0
pT2	259 (72%)	110 (77%)	96 (68%)	53 (69%)
pT3	100 (28%)	30 (21%)	46 (32%)	24 (31%)
Pathological Nodal Stage				
pN0	187 (83%; N = 225)	80 (89%; N = 90)	70 (80%; N = 88)	37 (79%; N = 47)
pN1	38 (17%; N = 225)	10 (11%; N = 90)	18 (20%; N = 88)	10 (21%; N = 47)
pNx	137 (38%; N = 362)	53 (37%; N = 143)	54 (38%; N = 142)	30 (39%; N = 77)
Margin Status				
R0	227 (63%; N = 359)	85 (60%; N = 142)	91 (64%; N = 142)	51 (68%; N = 75)
R1	132 (37%; N = 359)	57 (40%; N = 142)	51 (36%; N = 142)	24 (32%; N = 75)
RX	3 (1%; N = 362)	1 (0.7%; N = 143)	0	2 (3%; N = 77)

## Data Availability

Dataset is available by author upon request.

## References

[1] International WCRF (2020). Prostate Cancer Statistics. https://www.wcrf.org/cancer-trends/prostate-cancer-statistics/.

[2] Hamdy F.C., Donovan J.L., Lane J.A., Metcalfe C., Davis M., Turner E.L., Martin R.M., Young G.J., Walsh E.I., Bryant R.J. (2023). Fifteen-Year Outcomes after Monitoring, Surgery, or Radiotherapy for Prostate Cancer. N. Engl. J. Med..

[3] Cao L., Yang Z., Qi L., Chen M. (2019). Robot-assisted and laparoscopic vs. open radical prostatectomy in clinically localized prostate cancer: Perioperative, functional, and oncological outcomes: A Systematic review and meta-analysis. Medicine.

[4] Moschovas M.C., Brady I., Noel J., Zeinab M.A., Kaviani A., Kaouk J., Crivellaro S., Joseph J., Mottrie A., Patel V. (2022). Contemporary techniques of da Vinci SP radical prostatectomy: Multicentric collaboration and expert opinion. Int. Braz. J. Urol..

[5] Patel V.R., Samavedi S., Bates A.S., Kumar A., Coelho R., Rocco B., Palmer K. (2015). Dehydrated Human Amnion/Chorion Membrane Allograft Nerve Wrap Around the Prostatic Neurovascular Bundle Accelerates Early Return to Continence and Potency Following Robot-assisted Radical Prostatectomy: Propensity Score-matched Analysis. Eur. Urol..

[6] Koizumi N.J., Inatomi T.J., Sotozono C.J., Fullwood N.J., Quantock A.J., Kinoshita S. (2000). Growth factor mRNA and protein in preserved human amniotic membrane. Curr. Eye Res..

[7] Tseng S.C. (2001). Amniotic membrane transplantation for ocular surface reconstruction. Biosci. Rep..

[8] Zhu Y.T., Li F., Zhang Y., Chen S.Y., Tighe S., Lin S.Y., Tseng S.C.G. (2020). HC-HA/PTX3 Purified From Human Amniotic Membrane Reverts Human Corneal Fibroblasts and Myofibroblasts to Keratocytes by Activating BMP Signaling. Investig. Ophthalmol. Vis. Sci..

[9] Gao M., Zhao H., Tian D., Yu K., Bai J., Dong R., Zhang G. (2013). [Experimental study on human amniotic membrane for repairing tendon sheath defect]. Zhongguo Xiu Fu Chong Jian Wai Ke Za Zhi.

[10] Snyder R.J., Shimozaki K., Tallis A., Kerzner M., Reyzelman A., Lintzeris D., Bell D., Rutan R.L., Rosenblum B. (2016). A Prospective, Randomized, Multicenter, Controlled Evaluation of the Use of Dehydrated Amniotic Membrane Allograft Compared to Standard of Care for the Closure of Chronic Diabetic Foot Ulcer. Wounds.

[11] Pacaccio D.J., Cazzell S.M., Halperin G.J., Kasper M.A., Neutel J.M., O’Carroll B.D., Reyzelman A.M. (2018). Human placental membrane as a wound cover for chronic diabetic foot ulcers: A prospective, postmarket, CLOSURE study. J. Wound Care.

[12] Singh R., Purohit S., Chacharkar M.P., Bhandari P.S., Bath A.S. (2007). Microbiological safety and clinical efficacy of radiation sterilized amniotic membranes for treatment of second-degree burns. Burns.

[13] Puyana S., Elkbuli A., Ruiz S., Bernal E., McKenney M., Lim R., Askari M., Mir H. (2019). The Use of Dehydrated Human Amniotic/Chorionic Membrane Skin Substitute in the Treatment of Pediatric Facial Burn. J. Craniofac. Surg..

[14] Fesli A., Sari A., Yilmaz N., Comelekoglu U., Tasdelen B. (2014). Enhancement of nerve healing with the combined use of amniotic membrane and granulocyte-colony-stimulating factor. J. Plast. Reconstr. Aesthet. Surg..

[15] Karaman M., Tuncel A., Sheidaei S., Senol M.G., Karabulut M.H., Deveci I., Karaman N. (2013). Amniotic membrane covering for facial nerve repair. Neural Regen. Res..

[16] Noël J., Mascarenhas A., Patel E., Reddy S., Sandri M., Bhat S., Moschovas M., Rogers T., Ahmed S., Stirt D. (2022). Nerve spare robot assisted laparoscopic prostatectomy with amniotic membranes: Medium term outcomes. J. Robot. Surg..

[17] Noël J., Ahmed S., Mascarenhas A., Stirt D., Moschovas M., Patel E., Reddy S., Bhat S., Rogers T., Patel V. (2023). Impact of human placental derivative allografts on functional and oncological outcomes after radical prostatectomy: A literature review. J. Robot. Surg..

[18] Razdan S., Bajpai R.R., Sanchez M.A. (2019). A matched and controlled longitudinal cohort study of dehydrated human amniotic membrane allograft sheet used as a wraparound nerve bundles in robotic-assisted laparoscopic radical prostatectomy: A puissant adjunct for enhanced potency outcomes. J. Robot. Surg..

[19] Ahmed M., Esposito M., Lovallo G. (2020). A single-center, retrospective review of robot-assisted laparoscopic prostatectomy with and without cryopreserved umbilical cord allograft in improving continence recovery. J. Robot. Surg..

[20] Elliott P.A., Hsiang S., Narayanan R., Bierylo J., Chang S.C., Twardowski P., Wilson T.G. (2021). Cryopreserved placental tissue allograft accelerates time to continence following robot-assisted radical prostatectomy. J. Robot. Surg..

[21] Alvim R.G., Hughes C., Somma A., Nagar K.K., Wong N.C., La Rosa S., Monette S., Kim K., Coleman J.A. (2019). The potential risk of tumor progression after use of dehydrated human amnion/chorion membrane allograft in a positive margin resection model. Ther. Adv. Urol..

[22] Peak T.C., Praharaj P.P., Panigrahi G.K., Doyle M., Su Y., Schlaepfer I.R., Singh R., Griend D.J.V., Alickson J., Hemal A. (2018). Exosomes secreted by placental stem cells selectively inhibit growth of aggressive prostate cancer cells. Biochem. Biophys. Res. Commun..

[23] Bustos M.A., Yokoe T., Shoji Y., Kobayashi Y., Mizuno S., Murakami T., Zhang X., Sekhar S.C., Kim S., Ryu S. (2023). MiR-181a targets STING to drive PARP inhibitor resistance in BRCA- mutated triple-negative breast cancer and ovarian cancer. Cell Biosci..

[24] Shoji Y., Yokoe T., Kobayashi Y., Murakami T., Bostick P.J., Shiloh Y., Hoon D.S., Bustos M.A. (2022). UBQLN4 promotes STING proteasomal degradation during cisplatin-induced DNA damage in triple-negative breast cancer. Clin. Transl. Med..

[25] Bauer D., Wasmuth S., Hennig M., Baehler H., Steuhl K.P., Heiligenhaus A. (2009). Amniotic membrane transplantation induces apoptosis in T lymphocytes in murine corneas with experimental herpetic stromal keratitis. Investig. Ophthalmol. Vis. Sci..

[26] He H., Li W., Tseng D.Y., Zhang S., Chen S.Y., Day A.J., Tseng S.C. (2009). Biochemical characterization and function of complexes formed by hyaluronan and the heavy chains of inter-alpha-inhibitor (HC*HA) purified from extracts of human amniotic membrane. J. Biol. Chem..

[27] He H., Zhang S., Tighe S., Son J., Tseng S.C.G. (2013). Immobilized heavy chain-hyaluronic acid polarizes lipopolysaccharide-activated macrophages toward M2 phenotype. J. Biol. Chem..

[28] Lambiase A., Sacchetti M., Bonini S. (2012). Nerve growth factor therapy for corneal disease. Curr. Opin. Ophthalmol..

[29] Iwao A., Saijo H., Nakayama T., Higashi A., Kashiyama K., Mitsutake N., Tanaka K. (2023). Fresh human amniotic membrane wrapping promotes peripheral nerve regeneration in PGA-collagen tubes. J. Plast. Surg. Hand Surg..

[30] Chen L., Song X., Yao Z., Zhou C., Yang J., Yang Q., Chen J., Wu J., Sun Z., Gu L. (2023). Gelatin nanofiber-reinforced decellularized amniotic membrane promotes axon regeneration and functional recovery in the surgical treatment of peripheral nerve injury. Biomaterials.

